# Effects of Edge Directions on the Structural Controllability of Complex Networks

**DOI:** 10.1371/journal.pone.0135282

**Published:** 2015-08-17

**Authors:** Yandong Xiao, Songyang Lao, Lvlin Hou, Michael Small, Liang Bai

**Affiliations:** 1 Science and Technology on Information Systems Engineering Laboratory, National University of Defense Technology, Changsha, Hunan, China; 2 School of Mathematics and Statistics, The University of Western Australia, Crawley, WA, Australia; Wake Forest School of Medicine, UNITED STATES

## Abstract

Recent advances indicate that assigning or reversing edge direction can significantly improve the structural controllability of complex networks. For directed networks, approaching the optimal structural controllability can be achieved by detecting and reversing certain “inappropriate” edge directions. However, the existence of multiple sets of “inappropriate” edge directions suggests that different edges have different effects on optimal controllability—that is, different combinations of edges can be reversed to achieve the same structural controllability. Therefore, we classify edges into three categories based on their direction: *critical*, *redundant* and *intermittent*. We then investigate the effects of changing these edge directions on network controllability, and demonstrate that the existence of more critical edge directions implies not only a lower cost of modifying inappropriate edges but also better controllability. Motivated by this finding, we present a simple edge orientation method aimed at producing more critical edge directions—utilizing only local information—which achieves near optimal controllability. Furthermore, we explore the effects of edge direction on the controllability of several real networks.

## Introduction

One of the most fundamental challenges in network science is to understand the impact of structural properties on network functionality. In part, this is because understanding the network structure is prerequisite to answering the challenging questions related to the system’s dynamics [1–3]. Structure and dynamics are always coupled and ultimately led to many remarkable advances in the past decade in such areas as epidemic spreading [4, 5], random walks [6, 7], cascade failure [8, 9], and synchronization [10, 11]. Questions concerning the management and control of complex networks are important in engineering [12], finance [13], biology [14] and other fields because the ultimate proof of our understanding of such complex systems and their basic principles is reflected in our ability to control [15–19] and ultimately observe them [20]. The framework for computing the controllability of complex networks proposed by Liu and Barabási [19] offers a general, rigorous, and well-understood means of investigating how we can control complex networks with the fewest input signals. Recent advances in the application of control theory to complex networks have shed new light on this problem [21–30]. The main finding has been that the application of external signals to some subset of nodes can cause a system to approach a desired state within finite time.

Obviously, controllability in general can be achieved through the control of only the subset of nodes termed driver nodes. The minimum driver node set can be efficiently identified using the Minimum Inputs Theorem of Ref. [19]. Based on this approach to the minimization of the driver node set, several advances in the optimization of network controllability have already been made, the most representative methods of which rely on adding edges [25, 31], rewiring edges [32] and assigning edge directions [33, 34]. Compared with the first two methods (which stem from the modification of the network structure), assigning edge directions to optimize network controllability appears to be the most effective from the perspective of designing a network with improved or even optimal controllability because it neither changes the original structure of the network nor incurs the external cost of adding links. By *edge orientation*, we refer to the process of assigning edge directions to undirected edges in an undirected network or (equivalently) reversing the directions of directed edges in a directed network. This method aims to approach optimal controllability without requiring any modification of the network structure, by which we mean that the presence or absence of a link between each pair of nodes remains unchanged. To this end, Xiao et al. [34] have systematically proposed the definition and solution for the edge orientation of optimal controllability (EOOC). Solving the EOOC problem requires constructing a corresponding network and transforming the problem into one of finding a maximum independent set (MIS) of this corresponding network.

For directed networks, there are certain “inappropriate” edge directions that may weaken the controllability of a network, i.e., so-called dilations and inaccessibility. First, we must identify all these “inappropriate” edge directions in a network and then reverse them. However, there are several different “inappropriate” direction sets for a given network, and the existence of multiple “inappropriate” direction sets suggests that different edges play different roles in optimal controllability. Previous work has focused on how to detect these “inappropriate” edge directions while ignoring the effects of edge direction on the optimal network controllability. In this work, we briefly review the definition of the EOOC and the framework in which to solve this problem. The remainder of this paper is organized as follows. We first classify all edge directions into three categories: critical edge directions, which always appear in the optimal direction set during the optimization process and are also called the skeleton of optimal controllability; redundant edge directions, which have no effect on optimization; and intermittent edge directions, which participate in some optimal direction sets but not others. In Ref. [29], Jia et al. also propose the similar definition of node classification to explore the role of individual nodes in acting as a driver node in all, some or none of the control configurations. However, though the definitions of node classification and edge classification are similar ideas, the research points do not have directed relations. It is because that we study the roles of edge direction when we assign them to approach the optimal controllability for a given network skeleton. Next, we study the correlation between critical edge directions and driver nodes and demonstrate that the existence of more critical edge directions in a network implies not only a lower cost of modifying inappropriate edges but also better controllability. Motivated by this finding, we analyze the structural properties of the three categories of edge directions and develop a simple edge orientation method based on the generation of more critical edge directions. The results of numerical experiments suggest that this method achieves a near-optimal optimization effect while requiring only local structural information. Finally, we apply the developed tool to a series of real networks and successfully explain why the structures of electronic circuit networks take advantage of controllability.

### Edge Orientation for Optimal Controllability

**Fig 1:**
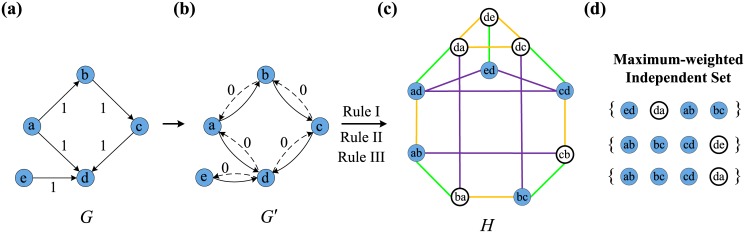
Illustration of Edge Orientation for Optimal Controllability. (a) A directed network *G* = (*V*, *E*) with 5 nodes and 5 directed edges. For directed networks, some edges with “inappropriate” direction affect the controllability of networks, such as *e*_*ed*_. If we change its direction, we can improve the controllability. (b) *G*′ is the symmetric directed network. Here we assume that the weight of real edge is 1 otherwise it is 0. Thus it results in the nodes of *H* with different weight. (c) The corresponding network *H*. The maximum-weighted independent set means not only the optimal edge orientation but also the least number of edges required to have their direction changed, because the node with weight 0 (the white nodes) in the maximum-weighted independent set implies the “inappropriate” edge of *G*. (d) The different maximum-weighted independent sets result in the different control configurations. The light blue nodes represent the real edges of *G*.

Ref. [34] provides a general solution to the problem of finding an edge direction set for optimal controllability, summarized as follows. First, construct a corresponding network and transform the EOOC problem into a problem of finding a maximum independent set of vertices in the corresponding network. A directed network *G* (Fig 1(a)) and its corresponding symmetric directed network *G*′ (Fig 1(b)) are a pair (*V*, *E*) and (*V*, *E*′) that consisted of a set of nodes *V* and edges *E*, *E*′. If each directed edge (*v*_*i*_, *v*_*j*_) in *G*′ corresponds to a node *a*_*ij*_, then we can construct a corresponding network *H* = (*A*, *B*) that consisted of a set of nodes *A* and edges *B*. The construction of links in *H* follows three simple principles.

- Rule I: two nodes in *H* that correspond to a pair of symmetric directed edges of *G*′ must be connected (green edges in Fig 1(c)).
- Rule II: two or more nodes in *H* that correspond to the edges pointing to a tail node must be connected (purple edges in Fig 1(c)).
- Rule III: two or more nodes in *H* that correspond to the edges pointing from a head node must be connected (orange edges in Fig 1(c)).

The next step is to calculate the maximum independent vertex set of *H*. Ref. [34] has shown that through the assignment of edge directions based on the MIS of *H*, the optimal controllability can be approached for a given undirected network. The detailed proof of this assertion can be found in Ref. [34]. In fact, the EOOC effectively mitigates the negative effects of inaccessibility and dilations, which are the two main factors that weaken network controllability.

In directed networks, the EOOC can allow for the detection of several “inappropriate” edges and the reversal of their directions to approach optimal controllability. For example, in Fig 1(a), the edge *e*_*ed*_ weakens the controllability because this directed edge results in the inaccessibility of node *e*. If we reverse the original direction of *e*_*ed*_, the number of required driver nodes decreases from 2 to 1. The extent of the directed network for the EOOC only considers the edge weights of the original network. We assume a directed graph *G* = (*V*, *E*, *W*) and its symmetric directed graph *G*′ = (*V*, *E*′, *W*′), both of which are edge-weighted (*W*, *W*′ represent the edge weight set). The weight of any real edge is 1 if it exists in *G*′; otherwise, it is 0 (Fig 1(b)). Thus, every node of *H* = (*A*, *B*, *W*′) corresponds to weight 0 or 1. The maximum-weighted independent set (MWIS) of vertices in *H* corresponds to the orientation set for optimal controllability of the directed network *G*. The basic theory follows from the analysis of the EOOC for an undirected network. The principle of maximum weight guarantees that the independent set contains the most nodes of weight 1 and the fewest nodes of weight 0, meaning that the number of edges that need to be modified is minimal. For example, Fig 1(d) shows the MWIS of *H*. The white nodes represent the reversal of “inappropriate” edge directions in Fig 1(a). It has been proven that the calculation of the MWIS is a typical NP-hard problem in computational complexity theory [35–37]; thus, a polynomial-time algorithm to find MWIS solutions for arbitrary graphs is not feasible. In the following, we will proceed using an integer linear programming (ILP) formulation for MWIS computation, in which the optimal solution was calculated using the IBM ILOG CPLEX v.12.6. From *H* = (*A*, *B*, *W*′), the ILP instance is constructed as following:

$$\begin{array}{ccc} \max\; & Z=\sum w_{i}^{\prime }x_{i} & \\ \text{s.t.}\; & x_{i}+x_{j}\leq 1,\forall \left(v_{i},v_{j}\right)\in B\\ & x_{i}=\left\{0,1\right\},v_{i}\in A \end{array}$$ (1)

The set {*v*∣*x*_*v*_ = 1} then clearly corresponds to a maximum-weighted independent set.

### Categories of Edge Directions for Optimal Structural Controllability

**Fig 2:**
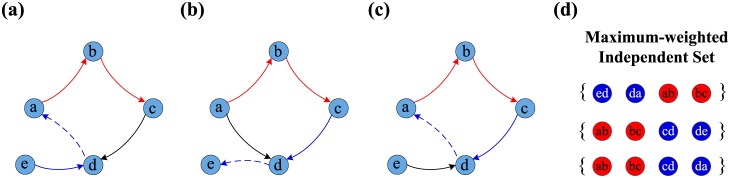
Illustration of classification of edge direction for EOOC in a directed network. (a, b, c) The networks are modified by maximum-weighted independent sets showing optimal controllability. (d) The different maximum-weighted independent sets show the different control configurations. The red nodes, *e*_*ab*_ and *e*_*bc*_, always appear on the MWIS, called the critical edge direction. The blue nodes, defined as intermittent edge directions, participate in some MWISs but not every MWIS.

A directed network possesses several sets of “inappropriate” edges because there are multiple MWIS configurations by which the network can approach optimal controllability. This phenomenon suggests that different edge directions have different effects on the optimal structural controllability. Therefore, the existence of these multiple configurations leads to the classification of edge directions into three categories: *critical edge directions*, corresponding to edges that are always present in the MWIS configurations; *redundant edge directions*, describing those edges that are never a part of an MWIS; and *intermittent edge directions*, corresponding to edges that participate in some MWISs but not in others. According to these definitions, edges that belong to the critical direction set play a significant role in the optimal controllability because they must appear in any optimal orientation set. Thus, the critical direction set can be regarded as the skeleton of optimal controllability. Conversely, the redundant direction set has no effect on the optimal controllability. The role of intermittent edge directions lies somewhere between these two extremes, as some cases require the directions of these edges to be reversed to approach optimal controllability. Fig 2 shows all optimal control configurations identified using the MWIS approach for Fig 1(a). The red edges in Fig 2(a), 2(b) and 2(c) and the red nodes in Fig 2(d) indicate the critical edge directions of the initial directed network *G*. The blue nodes in Fig 2(d) correspond to the intermittent edge directions. The blue dashed edge in Fig 2(a), 2(b) and 2(c) becomes an “appropriate” edge after the reversal of the original directions.

**Fig 3:**
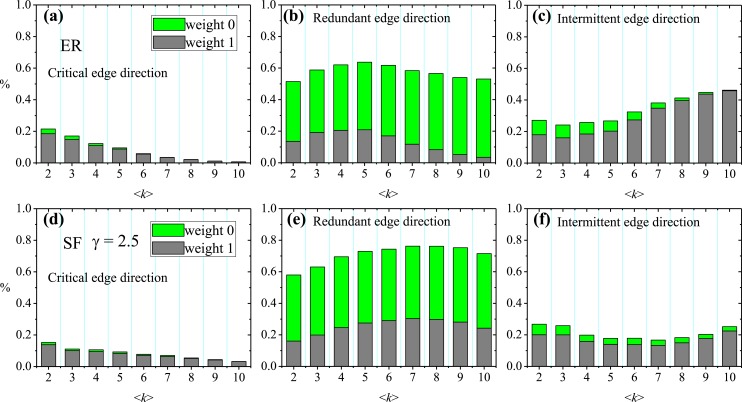
Characteristics of the fraction of three different edge direction sets in the directed networks. (a-c) ER network under different average degree 〈*k*〉. (d-f) SF network with *γ* = 2.5 under different average degree 〈*k*〉. The grey columns represent the real edges in the original directed networks *G* or nodes with weight 1 in the corresponding network *H*, while the green columns imply the non-real edges in *G* or nodes with weight 0 in *H*. The number of nodes is 10^3^.

The detailed procedures *Critical*MWIS (Algorithm 1 in S1 File) and *Redundant*MWIS (Algorithm 2 in S1 File) for the classification of edge directions are included in S1 File. Fig 3 shows the fractions of critical, redundant and intermittent edge directions in two basic network models of different average degree 〈*k*〉, the Erdös-Rényi (ER) model [38], and scale-free (SF) model [39]. SF networks are generated from the static model in Ref. [40]. Note that in the case of *γ* → ∞, this model is equivalent to the classical ER random network model. The green columns represent edges with weight 0, i.e., the non-real edges in the original directed networks. According to the definition of critical edge directions, the directions of edges with weight 0 (green columns) in the critical set must be modified during the optimization process. However, as 〈*k*〉 increases, the proportion of the critical set consisting of edges with weight 0 decreases and may even vanish. In the redundant edge direction set, non-real edges constitute the larger proportion, and as 〈*k*〉 increases, the redundant set tends to consist entirely of non-real edges. Compared with the redundant set, the intermittent edge direction set exhibits the opposite tendency of variation, as real edges constitute the larger proportion of this set. In particular, for an ER network with high 〈*k*〉, the intermittent set tends to consist entirely of real edges.

**Fig 4:**
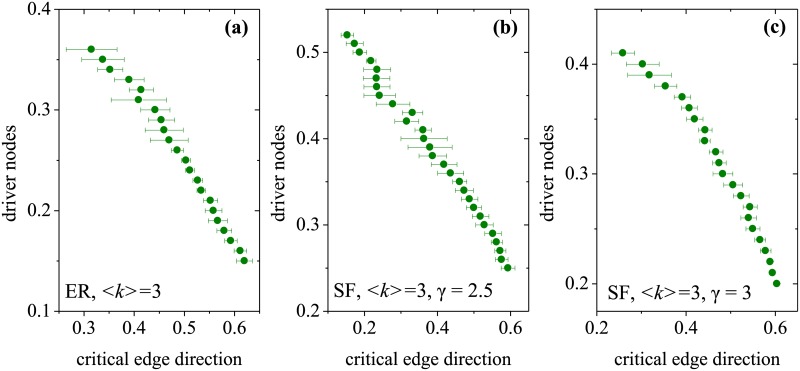
Scatterplot of the faction of critical edge directions the corresponding driver nodes. The number of nodes is 10^3^. We repeat this procedure until the total number of swapping reaches 10^6^. The results are 10 independent realizations.

In fact, the critical edge direction set can be regarded as the skeleton of optimal controllability because these directions can never be modified. In other words, these directions are always appropriate for a given network. If a network contains a larger number of critical edge directions, it implies not only a lower cost for modifying “inappropriate” edge directions but also better controllability. Therefore, we analyze the relationship between driver nodes and critical edge directions. Here, the fraction of driver nodes is a measure of the network controllability because the existence of fewer driver nodes implies better controllability. For a network with a fixed average degree, as shown in Fig 4, we consider various fractions of critical edge directions and their corresponding driver nodes in the ER and SF models. Here, a different fraction of critical edge directions in a given network is produced by edge swapping while keeping the degree of each node unchanged. Hence, in the original network, we swap the connections of two randomly chosen edges; that is, the edges *e*_*ij*_ and *e*_*kl*_ become edges *e*_*il*_ and *e*_*kj*_, respectively. At each step, we calculate and compare the fractions of critical edge directions before and after the edge swap. If the fraction of critical edge directions decreases, we consider the swap to be successful. If it is unsuccessful, the swap is reversed, and another swapping step is tested. As seen in Fig 4, for both ER and SF model, the number of driver nodes decreases with an increasing fraction of critical edge directions. The numerical results confirm the above analysis.

### Structural Analysis of Critical Edge Directions

**Fig 5:**
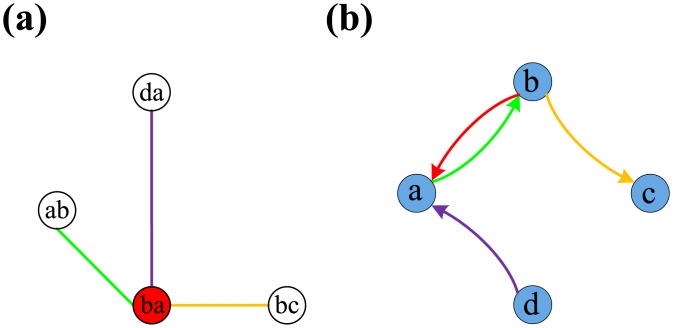
Illustration of three “inappropriate” control conditions. The red and green edges of (b) constitute a 2-cycle—which is meaningless for EOOC. The red and orange edges of (b) point from the common head node which produces dilation in the network. The red and purple edges of (b) point to the common tail node which increases an inaccessible node in the network. Actually, the corresponding degree of directed edge *v*_*ba*_ means the number of “inappropriate” control conditions.

To more deeply understand the properties of the three different edge direction sets, we analyze the corresponding degrees of edges with critical, redundant and intermittent edge directions. Here, the corresponding degree of an edge means its number of neighboring nodes in the corresponding network *H*, as every directed edge corresponds to a node in *H*. Fig 5(a) and 5(b) reproduce portions of Fig 1(c) and 1(b), respectively, to illustrate the meaning of the corresponding degree of an edge. The four nodes in Fig 5(a) correspond to the four edges in Fig 5(b), according to the rules of EOOC. In fact, these four edges in Fig 5(b) represent three “inappropriate” control conditions: two edges pointing toward the common tail (red and purple edges), two edges pointing from the head node (red and orange edges) and two edges constituting a 2-cycle (red and green edges). The first two “inappropriate” control conditions, called inaccessibility and dilation, respectively, can weaken controllability and should appear in a network as rarely as possible. The last condition, edges constituting a 2-cycle, is meaningless with regard to edge orientation because EOOC only assigns a single direction for an undirected link. According to the rules of link construction in *H*, the node in Fig 5(a) is connected with other nodes which produces the above three “inappropriate” conditions. Therefore, the corresponding degree of an edge reflects its associated number of “inappropriate” control conditions. Ref. [34] has deduced the node degree of the corresponding network *H*, i.e.,

$$k_{a(i,j)}=k_{a(j,i)}=k_{i}+k_{j}-1.$$ (2)

Here, *a*_(*i*, *j*)_ and *a*_(*j*, *i*)_ are the symmetric directed edges and correspond to two nodes in *H*, and *k*_*a*(*i*, *j*)_ is the corresponding degree of a directed edge.

**Fig 6:**
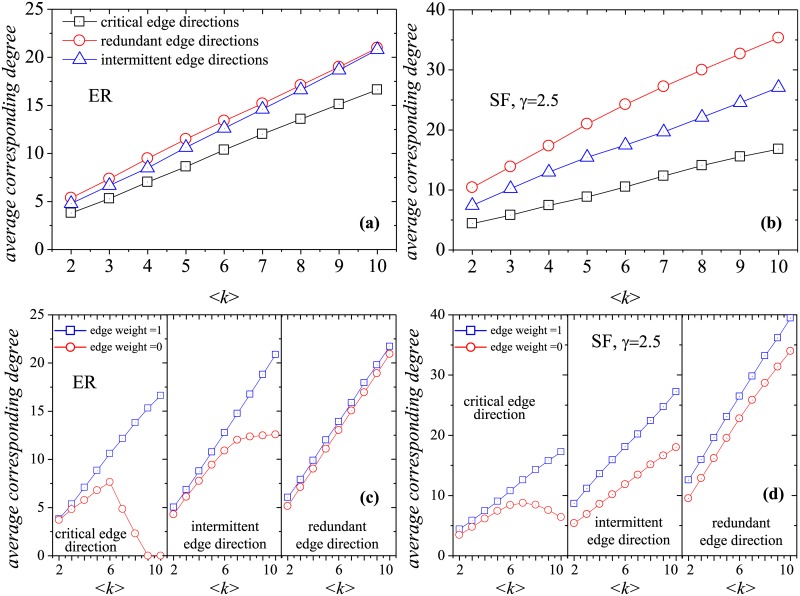
Characteristics of the corresponding degree of critical, redundant and intermittent edge direction set under different 〈*k*〉 for ER and SF networks. The corresponding degree of edge direction means the number of “inappropriate” control conditions. The subplots (c, d) display the degree of edge with weight 1 (real edges) and 0 (non-real edges) respectively. The number of nodes is 10^3^.

Fig 6 shows the average corresponding degrees of the critical, redundant and intermittent edge direction sets under various values of 〈*k*〉 for the ER and SF networks. The average corresponding degrees of the three direction categories represent the average numbers of associated “inappropriate” control conditions. In Fig 6(a) and 6(b), as 〈*k*〉 increases, the average corresponding degrees of all three categories also increase. However, compared with the other two direction sets, the critical direction set has the smallest average corresponding degree. Meanwhile, the redundant edge set has the largest average corresponding degree. The corresponding degree of the intermittent edge set is between those of the critical and redundant direction sets. Fig 6(c) and 6(d) display the corresponding degrees of edges with a weight of 1 (real edges) or 0 (non-real edges). For all three direction categories, the average corresponding degrees of real edges are larger than those of non-real edges. Moreover, the corresponding degree of critical edge directions associated with non-real edges initially increases and then decreases with increasing 〈*k*〉. It is because that, with the increase of 〈*k*〉, the fraction of critical edge directions corresponding to non-real edges become smaller and even disappeared, which could be approved by Fig 3(a) and 3(d). So these non-monotonic results are caused by the decrease in the number of critical edge directions associated with non-real edges. Therefore, an edge direction associated with fewer “inappropriate” control conditions is more likely to be a critical edge direction.

**Fig 7:**
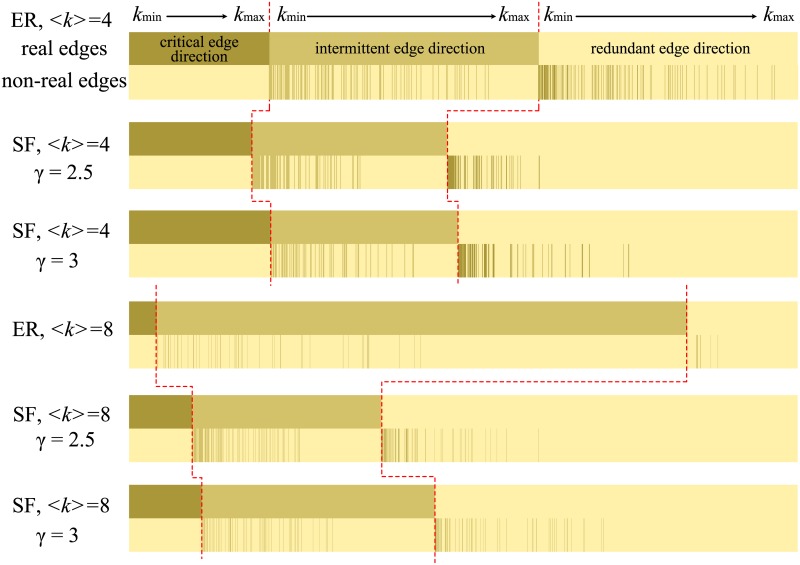
Characteristics of the category of symmetric real and non-real edges for ER and SF models. The x-axis displays every two symmetric directed edges. The real edges are divided into three categories and are ranked in ascending order of node degree. Due to the symmetric real and non-real edges having the same degree, the non-real edges are also ranked in the ascending order of degree. The number of nodes is 10^3^.

In Fig 7, we classify the real edges into three categories and rank the nodes in ascending order of corresponding degree. In accordance with the symmetric property of the corresponding network, we also display the category of each non-real edge. Thus, the *x* axis of Fig 7 refers to pairs of symmetric directed edges (real and non-real edges). Notably, the degrees of the symmetric non-real edges are also ranked in ascending order, and the two symmetric non-real edges have same degree. As seen from Fig 7, all symmetric non-real edges corresponding to edges in the critical edge direction set appear in the redundant edge direction set. This is because the critical edge directions appear in every MWIS and, therefore, their symmetric edges can never appear in any MWIS. With regard to the real edges associated with intermittent edge directions, their symmetric non-real edges of low degree also tend to belong to the category of intermittent edge directions, whereas most of their symmetric non-real edges of high degree are likely to appear in the redundant edge direction set. With regard to the real edges associated with redundant edge directions, their symmetric non-real edges of low degree tend to appear in the intermittent edge direction set or even the critical edge direction set, but their symmetric non-real edges of high corresponding degree are still likely to have redundant edge directions. These results indicate that nodes of low degree may have either significant (due to their critical edge directions) or partial (due to their intermittent edge directions) effects on optimal controllability.

### Edge Orientation by Critical Directions

In this section, we present a method for generating more critical edge directions, which we call *edge orientation by critical directions* (EOCD). The different categories of edge directions play different roles in the optimal controllability of a network. Based on the above analysis, we can draw two conclusions: (i) more critical edge directions are beneficial for enhancing controllability and decreasing the cost of improvements because there are fewer driver nodes and fewer “inappropriate” directions to be reversed, and (ii) an edge direction associated with fewer “inappropriate” control conditions is more likely to be a critical edge direction, that is, low-degree nodes have positive effects on optimal controllability. Moreover, previous studies of network controllability have indicated that the number of driver nodes is correlated with the control path [33] and the density of low in- and out-degree nodes [31]. In fact, the EOOC [34] approach attempts to produce the longest disjoint control path after the orientation of edge directions. Thus, we propose an edge orientation algorithm to produce the longest possible disjoint control path and as many critical edge directions as possible, starting from a node of the lowest degree. Consider an undirected network *G*(*V*, *E*). The EOCD method can be described as follows. The residual degree *k*′ of a node represents the number of undirected links of that node; *k*′ is initially equal to *k*, where *k* is the degree of the node. Note that if there are multiple such nodes matching the conditions, we randomly select one of them.

1. Compute *k*′(*i*) + *k*′(*j*) for two nodes *i* and *j* connected by a link *e*_*ij*_. Select the two nodes with the smallest sum of their residual degree. The selected node with the smaller degree is denoted as *V*_1_, and the selected node with big degree is labelled as *V*_2_. Note that we do not consider the isolated nodes.
2. Assign the edge direction to be from *V*_1_ to *V*_2_. Consequently, the residual degree of the selected nodes is reduced, i.e., *k*′(*V*_1_) = *k*′(*V*_1_) − 1, *k*′(*V*_2_) = *k*′(*V*_2_) − 1.
3. Select the node with the smallest degree among the neighbors of *V*_2_, and confirm that the newly selected node is not yet labelled. The newly selected node is denoted by *V*_2_, and the node formerly labeled *V*_2_ is now re-labelled as *V*_1_. Note that *V*_1_ and *V*_2_ are different.
4. Repeat step (ii) until all neighbors of *V*_2_ are labeled.
5. If *k*′ is equal to 0 for all nodes, proceed to step (vi). Otherwise, clear the labels all nodes, delete the assigned edges, and repeat step (i).
6. Orient all edge directions assigned at step (ii).

**Fig 8:**
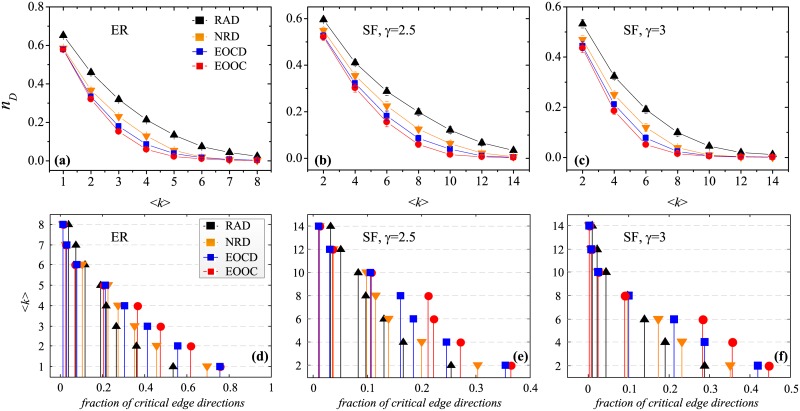
Characteristics of fraction of driver nodes (*n*_*D*_) and critical edge directions as a function of 〈*k*〉 for the method of random direction assignment (RAD), the method based on node residual degree (NRD), the method of edge orientation for optimal controllability (EOOC) and the method of edge orientation by critical directions (EOCD) on (a, d) ER networks, (b, e) SF networks with *γ* = 2.5, (c, f) SF networks with *γ* = 3. The number of nodes is 10^3^.

For comparative experiments, we consider the method of random direction assignment (RAD), a method based on the node residual degree (NRD) [33] and the method of edge orientation for optimal controllability (EOOC) [34]. Assigning directions based on the node residual degree (NRD) is an effective method of enhancing network controllability that is superior to random direction assignment (RAD). However, the NRD method cannot consider the effects of critical edge directions. The EOCD method is similar to the NRD method except for the manner in which the starting node is selected for each orientation step. Fig 8 (a), 8(b) and 8(c) present the optimization results of EOCD, which are close to the optimal EOOC solutions and superior to the NRD results. Fig 8 (d), 8(e) and 8(f) indicate that EOCD can generate more critical edge directions than the NRD method but, naturally, fewer than the EOOC. Moreover, the EOCD method is advantageous in terms of time complexity because it neither constructs a corresponding graph nor calculates the maximum independent set. In other words, EOCD requires only local structural information but achieves a near-optimal result.

### Analysis of Real Networks

**Fig 9:**
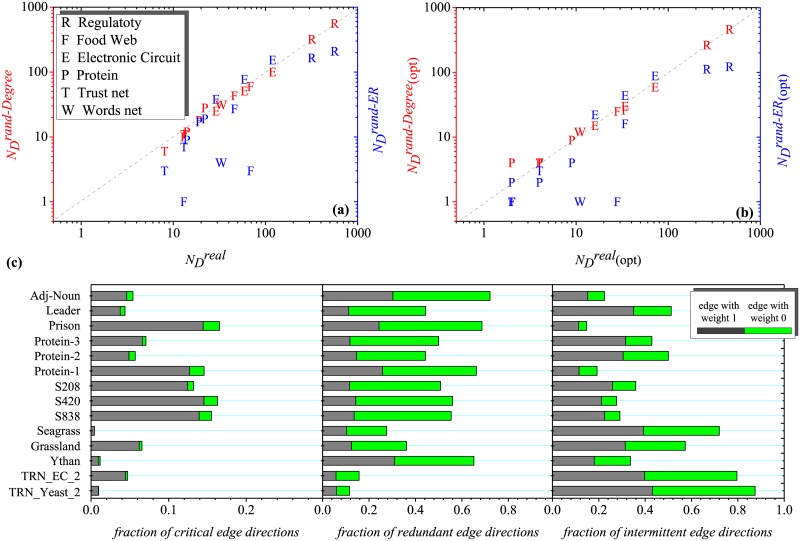
Plot of drive nodes of real, rand-Degree and rand-ER network (a) before ($n_{D}^{real}$, $n_{D}^{rand-Degree}$, $n_{D}^{rand-ER}$) and (b) after optimization by EOOC ($n_{D}^{real}\left(opt\right)$, $n_{D}^{rand-Degree}\left(opt\right)$, $n_{D}^{rand-ER}\left(opt\right)$), and (c) the faction of critical, redundant and intermittent edge direction of 14 real networks.

We now use the tools developed above to explore the controllability of several real networks, presented in Table 1. Previous work [19] has shown that there is no correlation between the *N*_*D*_ of an original network and the *N*_*D*_ of its randomized counterpart because a system’s controllability is, to a great extent, encoded by the underlying network’s degree distribution. To identify the topological features that affect the optimization of network controllability, we randomize each real network using a full randomization procedure (rand-ER) that transforms the network into a directed random ER network with *N* and *L* unchanged. We also apply a degree-preserving randomization procedure (rand-Degree), which leaves the in-degree and out-degree of each node unchanged but randomly selects which nodes are linked to each other. From the results presented in Fig 9(b), we find that the optimization is also affected by the network structure because $n_{D}^{real}(opt)$ is similar to $n_{D}^{rand-Degree}(opt)$. In fact, it can be said that the EOOC optimizes the controllability without changing the network skeleton. Moreover, we find that the networks of electronic circuits possess features distinct from those of other real networks. Generally, $n_{D}^{rand-ER}<n_{D}^{rand-Degree}\approx n_{D}^{real}$, because random networks are more easily controlled. However, the $n_{D}^{rand-Degree}$ of an electronic circuit network is smaller than $n_{D}^{rand-ER}$, and its $n_{D}^{rand-Degree}(opt)$ is also smaller than $n_{D}^{rand-ER}(opt)$. These results imply that the structure of an electronic circuit network is highly controllable. Meanwhile, compared with the other networks shown in Fig 9(c), the three electronic circuit networks possess a high number of critical edge directions, which can be regarded that the main skeleton of optimal controllability in these network is higher than other real networks. We also present the degree distributions of the electronic circuit networks in Table 2. Nodes of low degree (*k*_*in*_ or *k*_*out*_ = 1 or 2) account for a large proportion of the degree distribution. The number of driver nodes is determined be the density of low in- and out-degree [31]. Obviously, according to this conclusion, the electronic circuit networks should exhibit poor controllability. However, the true situation is precisely the opposite. The results indicate that the structures of the three electronic circuit networks is beneficial for structural controllability, and in the case of same average degree they require the fewer number of driver nodes.

**Table 1:** The characteristics of the real networks analyzed in the paper. For each network, we show its type, name; number of nodes (*N*) and links (*L*); driver nodes of real network $n_{D}^{real}$; driver nodes of real network after optimization $n_{D}^{real}(opt)$; driver nodes of rand-Degree network $n_{D}^{rand-Degree}$; driver nodes of rand-Degree network after optimization $n_{D}^{rand-Degree}(opt)$; driver nodes of rand-ER network $n_{D}^{rand-ER}$; driver nodes of rand-ER network after optimization $n_{D}^{rand-ER}(opt)$ respectively. All the data used are publicly available in the network databases from Ref. [42].

Type	#	Name	*N*	*L*	$n_{D}^{real}$	$n_{D}^{real}(opt)$	$n_{D}^{rand-Degree}$	$n_{D}^{rand-Degree}(opt)$	$n_{D}^{rand-ER}$	$n_{D}^{rand-ER}(opt)$
Regulatory	1	TRN_YEST_2	688	1069	0.821	0.670	0.811	0.669	0.303	0.177
	2	TRN_EC_2	423	519	0.754	0.620	0.755	0.623	0.388	0.264
Food Web	3	Ythan	135	601	0.511	0.207	0.438	0.185	0.017	0.003
	4	Grassland	89	137	0.523	0.375	0.479	0.285	0.297	0.176
	5	Seagrass	49	226	0.265	0.041	0.201	0.009	0.016	0.001
Electronic Circuits	6	S838	512	819	0.232	0.139	0.195	0.114	0.298	0.173
	7	S420	252	399	0.234	0.135	0.199	0.116	0.304	0.174
	8	S208	122	189	0.238	0.131	0.203	0.120	0.306	0.180
Protein	9	Protein_1	95	213	0.190	0.032	0.181	0.032	0.171	0.075
	10	Protein_2	53	123	0.245	0.038	0.224	0.059	0.161	0.064
	11	Protein_3	99	212	0.222	0.091	0.275	0.090	0.190	0.090
Trust	12	Prison	67	182	0.194	0.060	0.156	0.053	0.103	0.038
	13	Leader	32	96	0.219	0.031	0.180	0.005	0.072	0.023
Words	14	Adj_Noun	112	425	0.295	0.098	0.274	0.105	0.034	0.009

**Table 2:** The degree distribution of Electronic Circuits networks.

Name	〈*k*〉	in-degree		out-degree
		*P*(*k*_*in*_ = 1)	*P*(*k*_*in*_ = 2)	*P*(*k*_*out*_ = 1)
**S838**	3.199	0.371	0.473	0.768
**S420**	3.167	0.373	0.468	0.766
**S208**	3.098	0.377	0.459	0.762

However, of the real networks considered in this paper, the Prison and Protein-1 networks also have high fractions of critical edge directions; why are these two networks unable to demonstrate such excellent controllability? This can be explained in terms of *P*(*k*_*in*_ = 0). Nodes with *k*_*in*_ = 0 are called source nodes [41] because they must be controlled by input signals, that is, these nodes are driver nodes. In Table 3, it is seen that the *P*_*rand* − *ER*_(*k*_*in*_ = 0) values of S838, S420 and S208 are much larger than *P*_*real*_(*k*_*in*_ = 0), whereas the *P*_*rand* − *ER*_(*k*_*in*_ = 0) values of the Prison and Protein-1 networks are nearly equal to *P*_*real*_(*k*_*in*_ = 0). Here, *P*_*rand* − *ER*_(*k*_*in*_ = 0) is calculated using $P(k_{in})=P(k_{out})=e^{-z_{0}}z_{0}^{k}/k!$ with *z*_0_ = *z*/2 = 〈*k*_*out*_〉 = 〈*k*_*in*_〉. In the three real electronic circuit networks, the low *P*_*real*_(*k*_*in*_ = 0) values contribute to the fewer driver nodes. By contrast, in rand-ER networks with fixed average degree, the high *P*_*rand* − *ER*_(*k*_*in*_ = 0) values give rise to more driver nodes. Therefore, from the perspective of *P*(*k*_*in*_ = 0) and critical edge directions, we can fully explain why the structures of electronic circuit networks are more advantageous with regard to controllability than those of any other real networks.

**Table 3:** The *P*(*k*_*in*_ = 0) of five real networks.

	S838	S420	S208	Prison	Protein-1
〈*k*〉	3.199	3.167	3.098	5.433	4.484
*P*_*real*_(*k*_*in*_ = 0)	0.066	0.071	0.082	0.060	0.116
*P*_*rand* − *ER*_(*k*_*in*_ = 0)	0.202	0.205	0.213	0.066	0.106

## Discussion

In a directed network, the edge directions are the most significant properties that represent the ubiquitous dynamics of real networks. The edge directions and their correlations influence the driver nodes and are therefore especially relevant to the controllability of complex networks. Previous work has focused on developing edge orientation solutions to achieve the optimal controllability without changing the network structure, and this approach is more economical and practical than other optimization methods, such as adding or rewiring edges. However, it still lacks the ability to reveal the effects of different edge directions on optimal controllability, which could aid in understanding how the network structure affects controllability. For example, the structures of electronic circuit networks are advantageous for controllability despite the vast majority of nodes in these real networks having *k*_*in*_ and *k*_*out*_ values of 1 or 2. Based on the conclusion of previous work, controllability is determined by the density of low in- and out-degree nodes [31]. It is obvious that the electronic circuit networks should exhibit poor controllability. However, the true situation is precisely the opposite. Moreover, through degree-preserving randomization, the controllability of electronic circuit networks decreases noticeably. These behaviors can be explained by observing that these networks contain high concentrations of critical edge directions for optimal controllability. In summary, this work reveals the effects of the different classes of edge directions on optimal controllability and presents a simple edge orientation method based on the properties of the different direction sets. This simple method requires only local information but achieves a near-optimal optimization effect. All results demonstrate that the effects of edge directions can be recognized as a complement to previous measures in understanding network control.

## Supporting Information

S1 File The detailed procedures to computations of *Critical*MWIS (Algorithm 1) and *Redundant*MWIS (Algorithm 2). (PDF)

## References

[1] Barabási AL, Albert R (1999) Emergence of scaling in random networks. Science 286: 509–512. 10.1126/science.286.5439.509 10521342

[2] Newman M (2003) The structure and function of complex networks. SIAM Review 45: 167–256. 10.1137/S003614450342480

[3] Boccaletti S, Latora V, Moreno Y, Chavez M, Hwang DU (2006) Complex networks: Structure and dynamics. Physics Reports 424: 175–308. 10.1016/j.physrep.2005.10.009

[4] Moreno Y, Nekovee M, Pacheco AF (2004) Dynamics of rumor spreading in complex networks. Phys Rev E 69: 066130 10.1103/PhysRevE.69.066130 15244690

[5] Brockmann D, Helbing D (2013) The hidden geometry of complex, network-driven contagion phenomena. Science 342: 1337–1342. 10.1126/science.1245200 24337289

[6] Noh JD, Rieger H (2004) Random walks on complex networks. Phys Rev Lett 92: 118701 10.1103/PhysRevLett.92.118701 15089179

[7] Rosvall M, Bergstrom CT (2008) Maps of random walks on complex networks reveal community structure. Proc Natl Acad Sci USA 105: 1118–1123. 10.1073/pnas.0706851105 18216267 PMC2234100

[8] Motter AE (2004) Cascade control and defense in complex networks. Phys Rev Lett 93: 098701 10.1103/PhysRevLett.93.098701 15447153

[9] Buldyrev S V, Parshani R, Paul G, Stanley HE, Havlin S (2010) Catastrophic cascade of failures in interdependent networks. Nature 464: 1025–1028. 10.1038/nature08932 20393559

[10] Donetti L, Hurtado PI, Munoz MA (2005) Entangled networks, synchronization, and optimal network topology. Phys Rev Lett 95: 188701 10.1103/PhysRevLett.95.188701 16383953

[11] Arenas A, Díaz-Guilera A, Kurths J, Moreno Y, Zhou C (2008) Synchronization in complex networks. Physics Reports 469: 93–153. 10.1016/j.physrep.2008.09.002

[12] Sorrentino F, Bernardo M, Garofalo F, Chen G (2007) Controllability of complex networks via pinning. Phys Rev E 75: 046103 10.1103/PhysRevE.75.046103 17500957

[13] Delpini D. et al (2013) Evolution of controllability in interbank networks. Scientific Reports 3: 1626 10.1038/srep01626 23568033 PMC3620902

[14] Wuchty S (2014) Controllability in protein interaction networks. Proc Natl Acad Sci USA 111: 7156–7160. 10.1073/pnas.1311231111 24778220 PMC4024882

[15] Kalman RE (1963) Mathematical description of linear dynamical systems. Journal of the Society for Industrial & Applied Mathematics, Series A: Control 1: 152–192. 10.1137/0301010

[16] Lin CT (1974) Structural controllability. IEEE Transactions on Automatic Control 19: 201–208. 10.1109/TAC.1974.1100557

[17] Rugh WJ (1996) Linear System Theory (Prentice-Hall, Englewood Cliffs, NJ) 2nd ed.

[18] Lombardi A, Hörnquist M (2007) Controllability analysis of networks. Physical Review E 75: 056110 10.1103/PhysRevE.75.056110 17677136

[19] Liu YY, Slotine JJ, Barabási AL (2011) Controllability of complex networks. Nature 473: 167–173. 10.1038/nature10011 21562557

[20] Liu YY, Slotine JJ, Barabási AL (2013) Observability of complex systems. Proc Natl Acad Sci USA 110: 2460–2465. 10.1073/pnas.1215508110 23359701 PMC3574950

[21] Gao J, Liu YY, D’Souza RM, Barabási AL (2014) Target control of complex networks. Nature Communication 5: 5415 10.1038/ncomms6415 PMC4243219 25388503

[22] Yuan ZZ, Zhao C, Di ZR, Wang WX, Lai YC (2013) Exact controllability of complex networks. Nature Communication 4: 2447 10.1038/ncomms3447 PMC3945876 24025746

[23] Nepusz T, Vicsek T (2012) Controlling edge dynamics in complex networks. Nature Physics 8: 568–573. 10.1038/nphys2327

[24] Liu YY, Slotine JJ, Barabási AL (2012) Control centrality and hierarchical structure in complex networks. PLoS ONE 7: e44459 10.1371/journal.pone.0044459 23028542 PMC3459977

[25] Wang WX, Ni X, Lai YC, Grebogi C (2012) Optimizing controllability of complex networks by minimum structural perturbations. Phys Rev E 85: 026115 10.1103/PhysRevE.85.026115 22463287

[26] Hou LL, Small M, Lao SY (2014) Maximum entropy networks are more controllable than preferential attachment networks. Phys Lett A 378: 3426–3430. 10.1016/j.physleta.2014.09.057

[27] Pósfai M, Liu YY, Slotine JJ, Barabási AL (2013) Effect of correlations on network controllability. Scientific Reports 3: 1067 10.1038/srep01067 23323210 PMC3545232

[28] Hou LL, Lao SY, Small M, Xiao YD (2015) Enhancing complex network controllability by minimum link direction reversal. Physics Letters A 379: 1321–1325. 10.1016/j.physleta.2015.03.018

[29] Jia T. et al (2013) Emergence of bimodality in controlling complex networks. Nature Communication 4: 2002 10.1038/ncomms3002 23774965

[30] Jia T, Barabási AL (2013) Control capacity and a random sampling method in exploring controllability of complex networks. Scientific Reports 3: 2354 10.1038/srep02354 23912679 PMC3733055

[31] Menichetti G, Dall’Asta L, Bianconi G (2014) Network controllability is determined by the density of low in-degree and out-degree nodes. Phys Rev Lett 113: 078701 10.1103/PhysRevLett.113.078701 25170736

[32] Hou LL, Lao SY, Bu J, Bai L (2013) Enhancing Complex Network Controllability by Rewiring Links. IEEE Inter. Conf. on Intelligent System Design and Engineering Applications 709–711.

[33] Hou LL, Lao SY, Liu G, Bai L (2012) Controllability and Directionality in Complex Networks. Chin Phys Lett 29: 108901 10.1088/0256-307X/29/10/108901

[34] Xiao YD, Lao SY, Hou LL, Bai L (2014) Edge orientation for optimizing controllability of complex networks. Phys Rev E 90: 042804 10.1103/PhysRevE.90.042804 25375546

[35] Gazmuri PG. Independent sets in random sparse graphs. Networks 14: 367–377. 10.1002/net.3230140302

[36] Balaji S, Swaminathan V, Kannan K (2010) A simple algorithm to optimize maximum independent set. Adv Model Optim 12: 107–118.

[37] Robson J M (1986) Algorithms for maximum independent sets. J Algorithms 7: 425–440. 10.1016/0196-6774(86)90032-5

[38] Erdős P, Rényi A (1960) On the evolution of random graphs. Publ Math Inst Hungar Acad Sci 5 17–61.

[39] Barabási AL, Albert R (1999) Emergence of scaling in random networks. Science 286: 509–512. 10.1126/science.286.5439.509 10521342

[40] Goh KI, Kahng B, Kim D (2001) Universal behavior of load distribution in scale-free networks. Phys Rev Lett 87: 278701 10.1103/PhysRevLett.87.278701 11800921

[41] Ruths J, Ruths D (2014) Control profiles of complex networks. Science 343: 1373–1376. 10.1126/science.1242063 24653036

[42] Network controllability datasets. *http://boseinst.ernet.in/soumen/Network_Controllability_Datasets.html*.

