# Supplementary material for: Effects of Edge Directions on the Structural Controllability of Complex Networks
Source: PLoS One. 2015 Aug 17;10(8):e0135282. doi: 10.1371/journal.pone.0135282 (PMC4539223; doi:10.1371/journal.pone.0135282)
Supplement: S1 File — (PDF) [file pone.0135282.s001.pdf]

Effects of Edge Directions on the Structural Controllability of Complex Networks

Yandong Xiao[1,*], Songyang Lao[1], Lvlin Hou[1,2], Michael Small[2], Liang Bai[1]

1 Science and Technology on Information Systems Engineering Laboratory, National University of Defense Technology, Changsha, Hunan, China,
2 School of Mathematics and Statistics, The University of Western Australia, Crawley, WA, Australia

*** ydxiao@nudt.edu.cn**

Supporting Information

The detailed procedures *Critical*MWIS and *Redundant*MWIS

The detailed procedures *Critical*MWIS and *Redundant*MWIS to compute the set of critical or redundant edge directions in the MWIS respectively are described in the following. Let M be an MWIS. The following is a procedure to compute the set of critical edge directions, denoted by C_{MWIS}. It is to be noted that $Z(M)$ and $Z(I_v)$ actually means the size of original MWIS and MWIS after adding a constraint $x_v \leq 0$. To test a node whether it belongs to *Critical*MWIS set or not, we add a constraint $x_v \leq 0$ that assumes node v must not be in an MWIS. If v is a critical node of MWIS, the added constraint could lead to the decrease of the size of MWIS set because this node should always appear in every MWIS. Thus the value Z of MWIS under this constraint must be less than that of the original one. Similarly, computing the redundant directions just changes the constraint of computing the critical directions. In *Redundant*MWIS procedure, it is to be noted that $x_v \geq 1$ for $v \in V - M$ puts a constraint that node $v \in V - M$ must be in an independent set. Actually, the redundant node should never appear in every MWIS. If we put this redundant node into MWIS, it could result into the decrease of size of MWIS set, and then the value Z of MWIS under this constraint must be less than that of the original one.

Algorithm 1 The procedure of computing critical edge directions *Critical*MWIS.

Let M be an MWIS for $H(A, B, W)$;
Let $Z(M)$ be the size of M;
$C_{MWIS} \leftarrow \emptyset$;
for all $v \in M$ **do**
  Make an ILP instance I_v by adding a constraint that $x_v \leq 0$;
  Let $Z(I_v)$ be the size of an MWIS obtained from I_v;
  if $Z(I_v) < Z(M)$ **then**
    $C_{MWIS} \leftarrow C_{MWIS} \cup v$;
  end if
end for
return C_{MWIS};

Algorithm 2 The procedure of computing redundant edge directions *Reduandant*MWIS.

Let M be an MWIS for $H(A, B, W)$;
Let $Z(M)$ be the size of M;
$R_{MWIS} \leftarrow \emptyset$;
for all $v \in V - M$ **do**
    Make an ILP instance I_v by adding a constraint that $x_v \geq 1$;
    Let $Z(I_v)$ be the size of an MWIS obtained from I_v;
    if $Z(I_v) < Z(M)$ **then**
        $R_{MWIS} \leftarrow R_{MWIS} \cup v$;
    end if
end for
return R_{MWIS};
